# Integrating Medication Leaflets Utilizing FHIR and an LLM-Based Question-Answer Pipeline in a Mobile Application

**DOI:** 10.1007/s10916-026-02370-9

**Published:** 2026-04-01

**Authors:** Katharina Kirchsteiger, Lisa Heiler, Markus Bödenler, Sten Hanke

**Affiliations:** 1 eHealth Institute, FH Jonneum GmbH – University of Applied Sciences, Graz, Austria; 2 BBMRI-ERIC, Graz, Austria

**Keywords:** Large Language Models, FHIR, Medication Leaflets, LLM evaluation, Mobile Application

## Abstract

In the European Union, all medications must include a paper-based medication leaflet providing essential information. However, challenges with paper leaflets have driven efforts toward digitization and standardization. This paper presents an automated extraction, digitization, and standardization pipeline for medication leaflet information, integrated into a mobile medication management application. The automated extraction is using an LLM-based question-answer pipeline. Extracted information was standardized through template-based mapping to FHIR resources, where LLM-structured content was integrated into profile-aligned templates and validated using the $validate operation. To assess pipeline performance, ten randomly selected medication leaflets were evaluated, with 36 extracted fields per leaflet scored binary for correctness, completeness, and format adherence. Errors were categorized into four types: assignment errors (separating coherent information), missing information, interpretation errors (handling ambiguous content), and false information (hallucination). Mean scores were calculated per question set to identify extraction patterns. Fields with clearly delimited information—such as indications, excipient ingredients, and dose form—achieved mean scores of 0.9 or higher, with 20 of 36 fields demonstrating perfect accuracy. However, complex nested sections showed substantially lower performance: precautions scored 0.20 (predominantly assignment errors), interactions 0.30 (assignment errors), and undesirable effects 0.40 (primarily missing information). These recurring errors indicate the pipeline requires refinement for heterogeneous structural patterns in nested information. Structural inconsistencies in information hierarchies most significantly impacted extraction accuracy for these clinical use cases. By addressing these challenges, this approach opens new pathways in healthcare information management for mobile health developers and healthcare IT professionals. The mobile application could potentially be used by patients taking different medications and aims to improve medication literacy and accessibility. This work combines information extraction and standardization processes, laying potential groundwork for extending the approach to other healthcare areas. Practical benefits include enhanced searchability, multi-system interoperability, and improved patient access to structured medication information through mobile applications.

## Introduction

Every medication registered and marketed in the European Union (EU) must provide a paper-based medication leaflet with essential information. These includes instructions for proper use, precautions, warnings, and other crucial information [1]. Content is strictly regulated (in Austria by the Austrian Medicinal Products Act - “Arzneimittelgesetz” (AMG) § 16). Understanding these complex medical texts requires medication literacy—the ability to obtain, understand, and use medication information safely and effectively [2]. The medication leaflet remains a vital source for ensuring patients have access to essential and regulated information.

However, paper leaflets have practical limitations: small font sizes making them hard to read, risk of being lost or discarded, and large size due to extensive content (often exceeding 2000 words), ultimately having ecological impact [3,4,5]. The pharmaceutical industry has therefore discussed transitioning to digital formats. Digital leaflets offer several benefits: simpler access to updated information, improved searchability through structured data, multi-system usability, and enhanced accessibility via features like zoom functionality and text-to-speech [5]. The European Medicines Agency’s (EMA) electronic product information (ePI) pilot project selected FHIR (Fast Healthcare Interoperability Resources) as the common standard for harmonizing ePI information [6]. FHIR is an HL7 standard for exchanging healthcare information electronically, leveraging modern web technologies like REST, XML, and JSON to provide flexible interoperability [7]. Data standardization, as explained by the FAIR principles, is essential for good data management [8], and studies have shown that FHIR can support FAIR-related aspects [9]. In addition, FHIR has proven beneficial for medical device connectivity, clinical data management, and patient information coordination [10,11].

Systematic reviews summarize Natural Language Processing (NLP) methods and their application to unstructured Electronic Health Records (EHR) data and healthcare decision-making [12,15]. Other work focuses on extracting real-world data variables from EHRs and demonstrates task-specific NLP pipelines on clinical notes [13,14]. More recently, Large Language Models (LLM) have been shown to encode clinically relevant knowledge and to support information extraction in biomedical domains, including medical case reports [16,17,18]. However, these works mainly focus on patient-centric clinical documents. Medication leaflets, by contrast, are regulated informational documents with a more standardized section structure and instruction-focused content. This leaves a gap in extracting and standardizing regulated, non-patient information from medication leaflets using FHIR.

This paper aims to develop and evaluate a prototype to perform information extraction from medication leaflets and subsequent standardization using FHIR, with the goal of providing accessible and structured medication information in a mobile application. The primary use case targets patients who seek access to leaflet information, while the underlying standardized representation is designed to support interoperability and reuse by developers and potentially other institutions. This work extends a previous prototype [19], called MediScan, by enabling more detailed extraction, a profile-driven FHIR representation aligned with AMG § 16 requirements, and end-to-end integration of standardized leaflet content into MediScan application.

## Methods

The methodology comprised (i) FHIR resource identification, (ii) profiling and Implementation Guide (IG) development, and (iii) system implementation. Back-end services were developed in Python utilizing fhir.py (v1.4.2) [20] and fhir.resources (v7.1.0) [21], managed by Poetry. Front-end used Flutter (v3.16.0) with Dart (v3.2.0) plus the Flutter/Dart package fhir (v0.12.0) [22]. GPT-4o was used for information extraction.

### FHIR Resource Identification, Profiling and IG Development

**Fig. 1:**
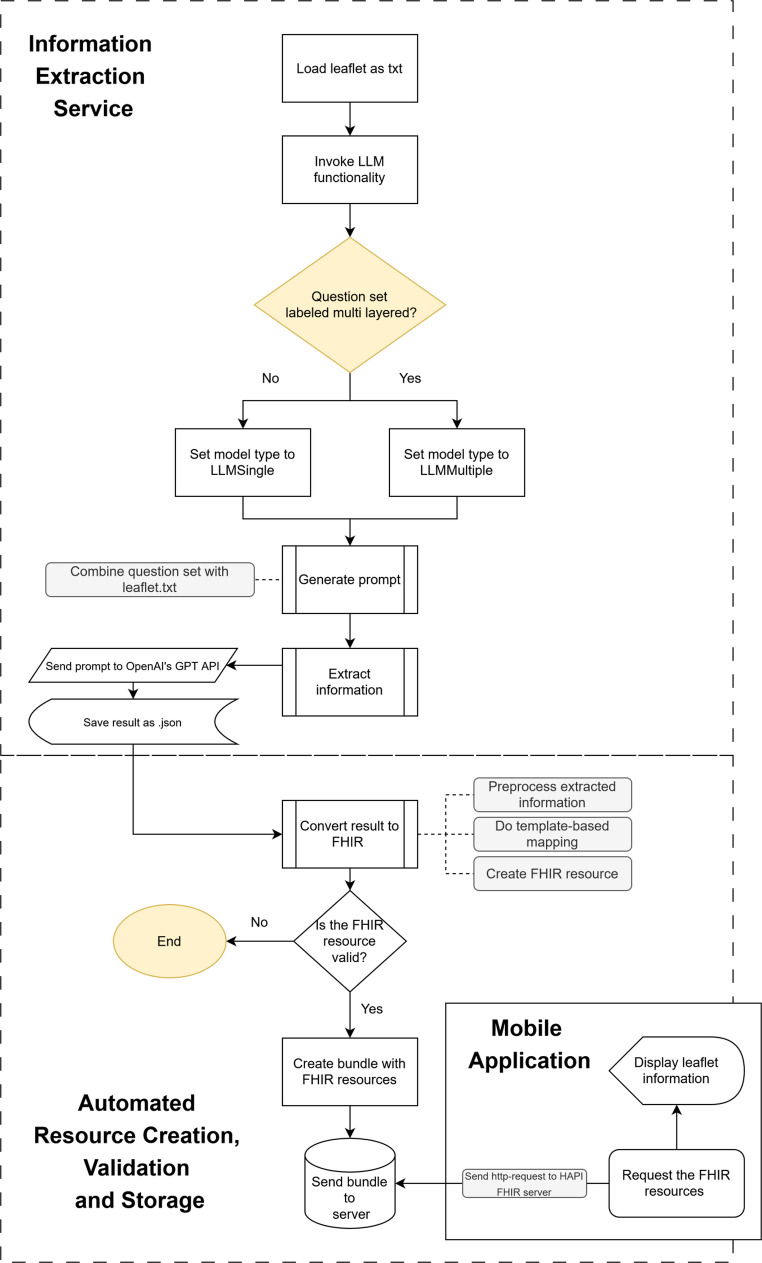
Visualization of the data processing pipeline. The upper half depicts the information extraction service—which loads leaflet text, selects the prompting mode (LLMSingle vs. LLMMultiple), builds and submits prompts to GPT, and saves structured answers—while the lower half shows automated FHIR conversion, validation, bundle creation, and storage on a HAPI FHIR server, from which the mobile app requests and displays the resources

At first, we derived mandatory content items by analyzing medication leaflets authorized in Austria and the AMG § 16 text. Each item was mapped to FHIR R5 by selecting semantically suitable resources and elements and documented the mapping in tables. Figure 1 depicts the AMG § 16 required content and the selected FHIR resource types. Alternative resources such as Medication (designed for administered medication instances), MedicationStatement (for patient history), and DocumentReference (document-centric rather than granular data access) were excluded to prioritize granular data access. Since base FHIR resources lack AMG § 16-specific constraints, custom profiling was used to ensure machine-validatable compliance. Based on the selected resources, we created FHIR profiles using StructureDefinition resources to constrain resource structure. Profiles and IG artifacts were authored in FHIR Shorthand (FSH) and rendered into browsable IG using IG-publisher [23]. The IG was not officially published on the web, as it was leveraged as a proof of concept for this specific use case. Technical validation was performed through successful compilation with IG-publisher.

Profiling was preferred over extensions because the required rules could be expressed using standard elements through cardinality constraints, slicing, and value restrictions. Extensions were not required because no additional data elements beyond the available FHIR structures were introduced.

### Extracting Information from a Medication Leaflet by Leveraging an LLM

We implemented an LLM-based question–answer pipeline that combines leaflet text with predefined question sets, submits prompts to GPT-4o, and stores the extracted answers as JSON key–value pairs (Fig. 1).

#### Model Integration and Question Set Definition

GPT-4o was used due to its context and output capacity and its ability to provide detailed and structured results [24]. A system role instruction (eHealth expert) required answers to be based only on leaflet content and to follow formatting rules (e.g., no cross-references or bullet points). Temperature was set to 0 for reproducibility.

Questions were authored manually as targeted extraction instructions that specified the requested information and the expected output format (e.g., enumeration, integer-only, or free text). Questions were bundled into five question sets of thematically related questions that jointly elicit all information required for a given FHIR resource or a defined information block. The questions refer to medication leaflet information and were formulated for orally administered medicines, as other administration routes require different questions.

Questions were either single-layered (direct extraction) or multi-layered. Multi-layered questions used a two-step ‘context pruning’ design: a high-level query first extracted the relevant subsection, followed by detailed extraction of specific items. This approach can be interpreted as LLM-based context pruning [25].

The approach is RAG-inspired in the sense of a retrieve-then-read-style pipeline, but the retrieval is performed within a single document (subsection extraction) rather than from an external source [26]. Table 1 shows example questions, their layer type, and the associated JSON keys.

**Table 1:** Example questions from the questions sets (English translation; original questions were authored in German), their layer type and the associated JSON key

Type	JSON key	Example extraction question (English translation)
Single-layered	forgotten	Search the leaflet for instructions on what to do if a dose is forgotten. Return only the instructions described without a heading.
Multi-layered (initial)	precautions	Search the leaflet for the chapter describing what must be considered before taking the medication. This chapter includes information on when the medication must not be taken, warnings and precautions, interactions with other medicines, and further warnings and notices. Extract the entire chapter including the section headings.
Multi-layered (follow-up)	precautions .contraindications	From the provided text, extract only the circumstances under which the medication must not be taken. Separate entries with | and use only information from this subsection and omit introductory headings.

#### LLM-Based Information Extraction Pipeline

The information extraction service represents the first step of the overall data processing pipeline (Fig. 1). It comprises two components. The first component performs orchestration and file handling: it loads the leaflet as a .txt file, iterates over predefined question sets, and selects the prompting strategy based on the question set type (single-layer vs. multi-layer). Strategy selection is implemented via two model classes, LLMSingle and LLMMultiple, which inherit from BaseLLM to ensure consistent role assignment while implementing different prompting behavior. The second component executes the LLM interaction reflected in Fig. 1 by generating the prompt from the question set and leaflet text, sending it to OpenAI’s GPT API, and persisting the returned answers as JSON. LLMSingle prompts each question directly against the full leaflet text, whereas LLMMultiple first extracts a relevant leaflet subsection and then prompts follow-up questions against that extracted text. Across five question sets, we defined 13 single-layer questions and six multi-layer questions with 23 follow-up questions, resulting in 42 prompt calls per leaflet. JSON keys serve as stable identifiers for downstream field mapping and enable traceability of each extracted value to its originating question.

#### Prompt Engineering

Iterative prompt engineering followed OpenAI guidelines [27]. Questions were tested on a development sample of six leaflets by comparing each response against the leaflet text as ground truth. If information was missing or misinterpreted, the corresponding question was revised and previously processed leaflets were re-run. The question sets were frozen once they were sufficiently stable for proof-of-concept evaluation to reduce the risk of overfitting to the development sample.

#### Evaluating the LLM’s Response

We evaluated the LLM output by manually comparing each extracted field against the medication leaflet text. Ground truth was implicitly derived from the leaflet text (no separate expert annotation). For each leaflet, the model produced a structured JSON output containing 36 predefined fields (JSON keys across five question sets) that were further processed into FHIR resources. Fifteen fields had predefined output formats, including list/enumeration fields and strict formats (e.g., integer-only, use of ; as separator). Each field received a binary score (1 correct, 0 incorrect). Binary scoring was used to provide a transparent and consistently applicable proof-of-concept assessment across heterogeneous field types, but it does not capture partial correctness. For list/enumeration fields, correctness required complete enumeration and semantically correct content for each item. For all other fields, outputs were labeled correct when they semantically aligned with the leaflet content and met the predefined format, if applicable. Semantic alignment was judged manually based on meaning equivalence rather than exact word matching. For incorrect fields, a short error note was recorded and failures were categorized into four error types (assignment error, missing information, interpretation error, false information). Ambiguous cases were rated conservatively as incorrect.

Ten distinct leaflets (list available in repository) were randomly selected from Austria-registered medications (AMPI database) after filtering for orally administered human medicines and excluding non-target categories (e.g., narcotics, topical products, homeopathic/phytopharmaceutical, veterinary, and non-oral routes). Given the proof-of-concept design and sample size (*n* = 10), the evaluation was intended to identify systematic error patterns rather than to provide statistically generalizable performance estimates.

Binary scores were aggregated into mean accuracies per question set by averaging field scores within each set and then averaging across leaflets. Mean accuracy per field was calculated by averaging its binary scores across the ten leaflets.

### Automated Creation and Validation of FHIR Resources

To create FHIR resources from extracted data, we implemented template-based mapping in which a profile-aligned template is defined per IG resource and assigns extracted JSON fields to the corresponding FHIR elements. Prior to mapping, selected fields are preprocessed (e.g., splitting enumerations and cleaning separators). Optional elements were omitted when no value was available; remaining nulls were set to “not applicable”, any other conflicts were not handled automatically. Each resource was validated individually by invoking the server-side $validate operation against the corresponding StructureDefinition. If validation failed for any resource, we logged the failure for the corresponding leaflet. While $validate does not perform semantic content checks, it enforces structural rules such as the presence of mandatory elements and references. Missing prerequisite resources could therefore cause downstream validation failures in dependent resources. Only resources that passed validation were combined into a transaction Bundle and sent to the HAPI FHIR server as an atomic commit, where the entire set succeeds or fails, to ensure data integrity. The workflow is shown in the lower part of Fig. 1.

### Integrating Standardized Medication Leaflet Information into MediScan

The developed IG guided tailoring the MediScan UI to medication leaflet content and served as the central specification for integrating the profiled FHIR structures into the application. A local FHIR server was deployed using the hapi-fhir-jpaserver-starter package [22] with PostgreSQL 13, both running via Docker. FHIR R5 was selected because the medication model requires R5-specific resources (e.g., MedicinalProductDefinition). MediScan retrieves resources via RESTful GET requests using the admission number (i.e., the national authorization identifier) to remain consistent with the AMPI database scheme. The _revinclude parameter is used to obtain ClinicalUseDefinition instances referencing a given MedicinalProductDefinition to reduce client-side requests. Retrieved resources are then rendered in MediScan to present the leaflet information.

## Results

Chapter 3 reports the selected FHIR resources, the derived AMG § 16-aligned profiles and IG, and findings on prompt engineering and extraction quality.

### Selected FHIR Resources

**Fig. 2:**
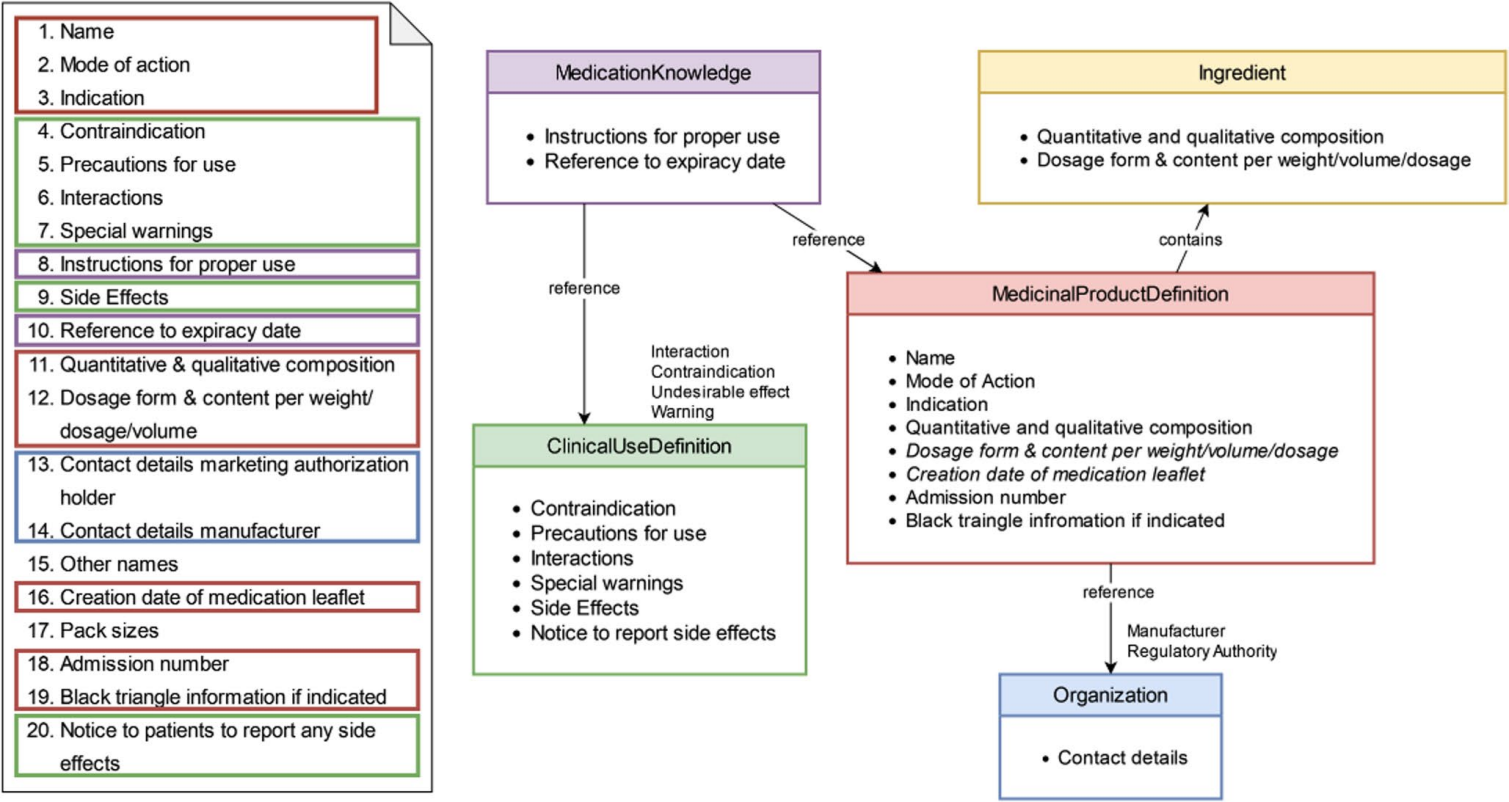
Visualization of the legally required content of medication leaflets according to AMG § 16 (left) and the corresponding FHIR resource types and relationships (right). Color coding links leaflet sections to their respective FHIR resources. The Ingredient resource is highlighted separately due to its inclusion within the MedicinalProductDefinition resource

Analysis of medication leaflet structure and official FHIR resources indicated that five resource types were sufficient to represent the required leaflet content (Fig. 2). The selected resources were MedicinalProductDefinition, Ingredient, ClinicalUseDefinition, MedicationKnowledge, and Organization, drawn from the Specialised, Clinical, and Base modules. Together, these resources captured all mandatory AMG § 16 elements within scope, with references preserving interlinked access to the full leaflet information across resources.

Two AMG § 16 elements were excluded. Alternative medication names were omitted because they appear inconsistently across leaflets, limiting reliable extraction. This has minimal impact for Austrian users but would be required for EU-wide deployment. Pack size was not modeled although mappable to PackagedProductDefinition, because robust product–package linkage was out of scope. This limits package-level use cases such as dispensing- or packaging-specific presentation. Both exclusions are scope limitations and could be addressed through extended profiling and improved extraction patterns.

### FHIR Profiling

Nine FHIR profiles were defined to encode AMG § 16-driven structural requirements, mainly cardinality rules and section-specific constraints. Multiple profiles were needed where leaflet content required clearly different structures. Ingredient was profiled separately for active ingredients and excipients because the required elements and constraints differ. ClinicalUseDefinition required four profiles to reflect distinct clinical sections: interactions, contraindications, undesirable effects, and warnings. Separate profiles were also created for MedicinalProductDefinition, MedicationKnowledge, and Organization. Slicing was applied where recurring elements needed distinct semantics. For example, slicing the dosingGuideline element in MedicationKnowledge separates missed-dose instructions from discontinuation guidance.

These nine profiles represent the minimal granularity needed for automated server-side validation of AMG § 16 constraints using the $validate operation. Fewer profiles would not allow section-specific constraints to be validated reliably. More profiles would increase complexity without adding regulatory benefit.

### A FHIR IG for Austrian Medication Leaflets

**Fig. 3:**
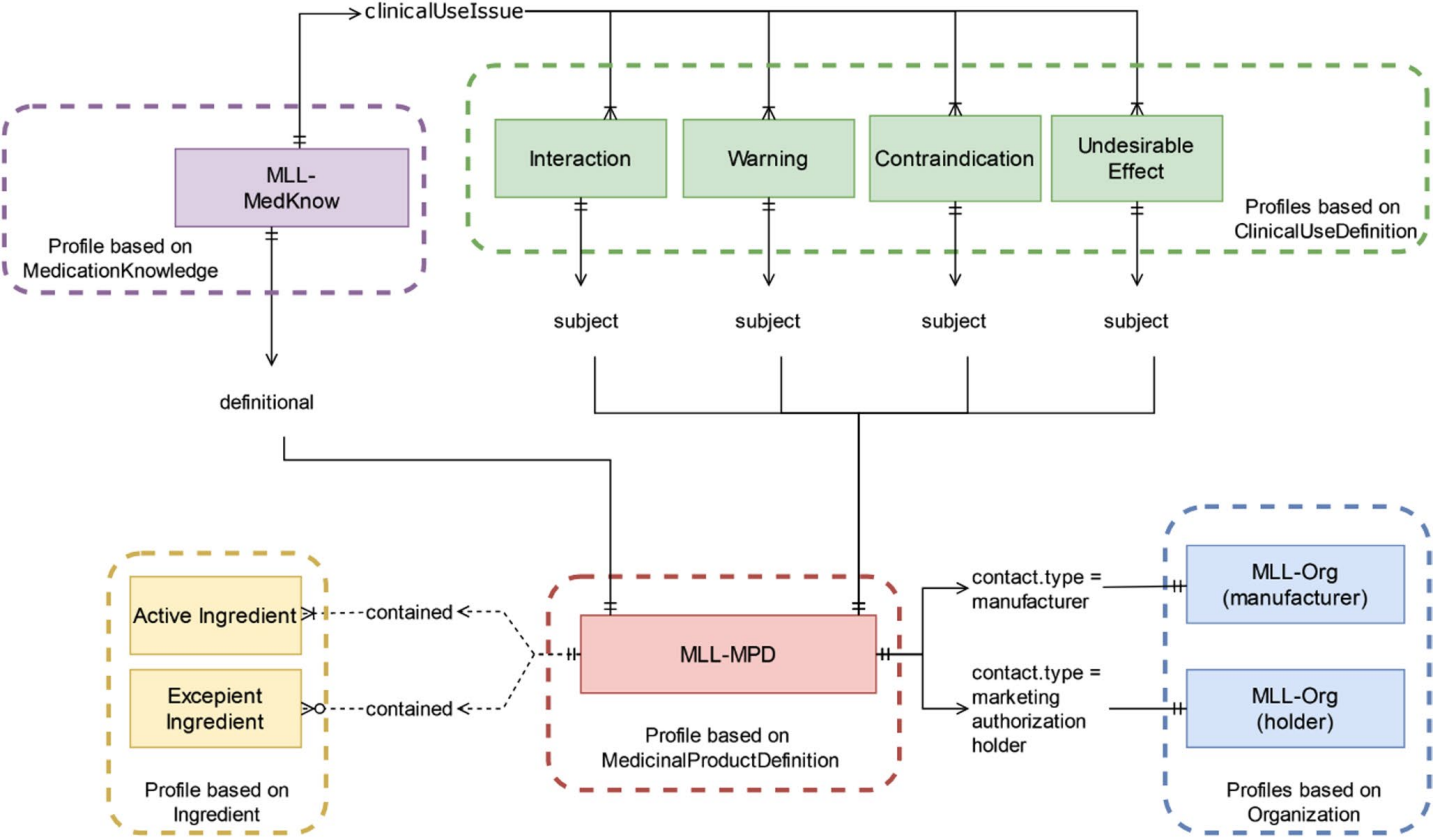
Visualization of the profiles used (colored rectangles), their parent resources (dashed rectangles), and their relationships. References are shown with solid lines, while dashed lines indicate contained resources. Cardinality is presented using UML notation on the arrows, the direction of the relation is shown by the arrow before the label. The color scheme corresponds to those in Fig. 2

The developed IG serves as a reusable specification for integrating the profiles into the MediScan UI and can be rebuilt from the repository’s FSH artifacts using SUSHI and the IG Publisher. Existing ePI-related IGs primarily model product information as documents using Composition as the main container. Whereas, our approach represents leaflet content as interconnected resources, with MedicinalProductDefinition as the entry point and related data retrieved via references and _revinclude. This enables granular, searchable access to leaflet information. The IG focuses on medication leaflet content rather than full ePI scope. Figure 3 summarizes the IG content and highlights the central role of the MLL-MPD (MLL=medication leaflet, MPD=MedicinalProductDefinition) profile.

### Prompt Engineering Findings

Prompt engineering was iteratively refined to improve extraction reliability and output formatting. Key findings were:

1. **Addressing Hallucination**: Some responses contained unsupported information. To mitigate hallucinations, prompts were updated to answer only when the content is present in the leaflet and otherwise return “None”.
2. **Output formatting**: Simple enumerations could be enforced with direct instructions. For complex structured lists, adding short in-prompt examples was necessary to achieve the required format .
3. **Token Efficiency**: Multi-layer prompts facilitated token optimization. As an illustrative example, the multi-layer setup (one high level question plus three follow-up questions) required 5643 tokens in total. Prompting the three follow-up questions separately with the full leaflet text required 15,873 tokens in total, corresponding to a 65% reduction.
4. **Multi-Layered-Question Sets**: Single-layer prompts were sufficient for well-delimited sections. Multi-layer prompting was applied for broad, nested sections such as warnings, precautions, interactions, and contraindications. Layer 1 restricted the prompt context to the relevant leaflet subsection, enabling more targeted follow-up questions in Layer 2.

### Evaluation of the LLM’s Response

Format-constrained fields showed no format deviations during manual inspection or automated processing, but content accuracy remained challenging, indicating that format compliance does not guarantee correct extraction. Based on notes for fields labeled “false”, errors were categorized into four types:

- **Error A (Assignment)**: Information was extracted but grouped incorrectly. For example, precautions for elderly patients and for patients with kidney disease were returned as separate items, although they belonged to a single “special populations” precaution block, which would lead to fragmented presentation after mapping.
- **Error M (Missing information)**: Leaflet-stated items were incompletely extracted. For instance, in the interactions medications field, warnings listed in nested bullet points or subsection lists were often missed, resulting in incomplete coverage.
- **Error I (Source ambiguity/ Interpretation)**: Some leaflets covered multiple product variants (e.g., different strengths with different admission numbers) in one document and listed variant-specific dosage instructions. Because the leaflet did not clearly link each dosage statement to a specific variant, extraction of a single correct dosage was inherently ambiguous.
- **Error F (False information)**: Unsupported content was generated. During early testing, the model produced dosage instructions for specific age groups that were not stated in the leaflet, which required stricter prompting.

**Fig. 4:**
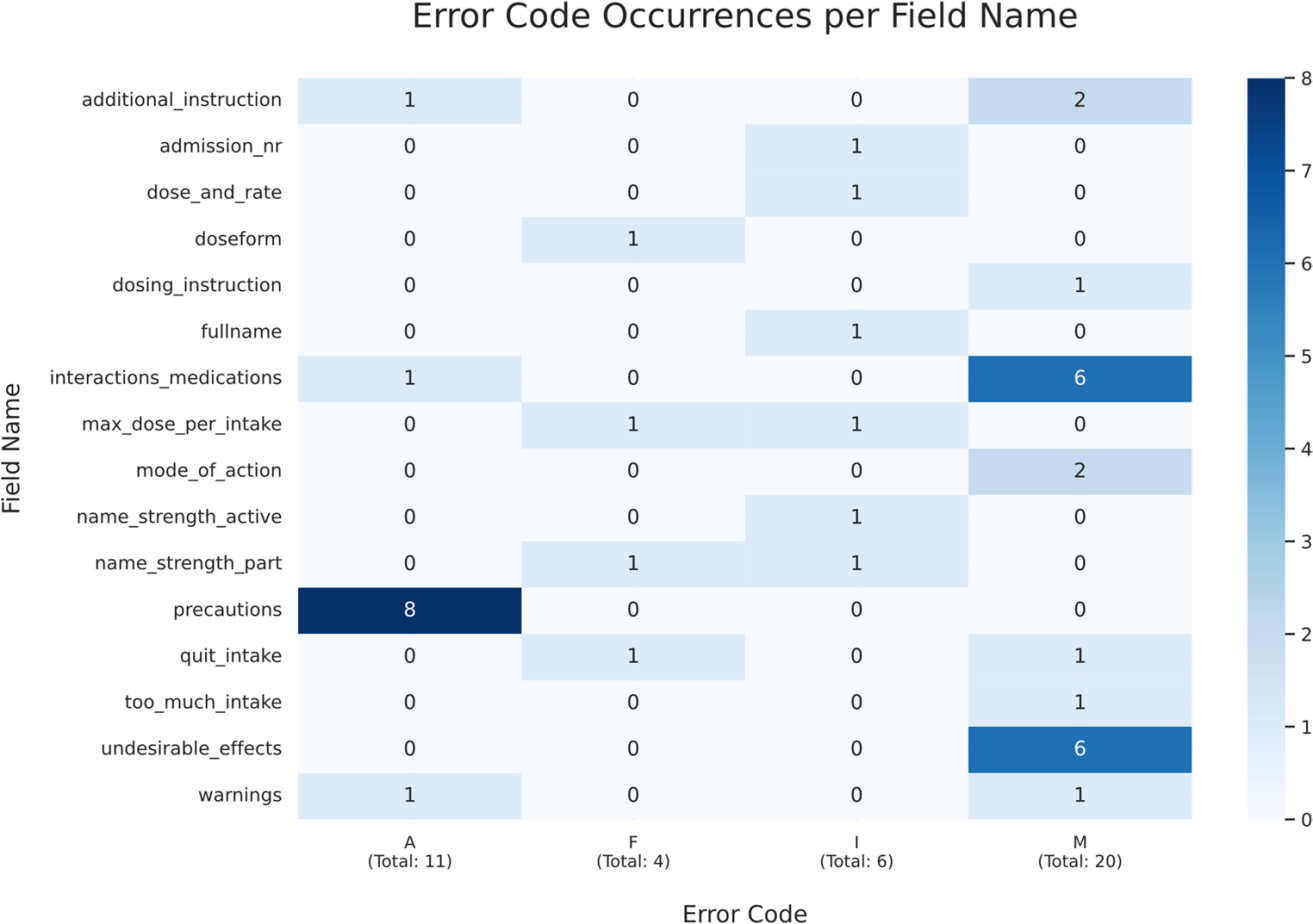
Heatmap depicting the distribution of error types (A: Assignment Errors, F: False Information, I: Interpretation Errors, M: Missing Information) across various fields. Occurrence is presented by color intensity. The total number of error types are listed in the labels for x-axis

Error F was primarily attributable to prompt design and was mitigated by instructing the model to return “None” when information was absent. Error A (Assignment) persisted in broad sections due to task complexity and prompt scoping. In contrast, Error M and Error I were predominantly driven by source and model limitations in heterogeneous or ambiguous leaflet sections, where relevant information is dispersed, nested, or not uniquely specified. Figure 4 summarizes the distribution of error types per field.

Fields with well-delimited information achieved scores of 0.9 or higher, whereas complex nested sections remained difficult (precautions 0.20, interactions 0.30) despite iterative refinement. At question-set level, ORG (extracting manufacturer information) achieved 1.0 and UNDES (extracting undesirable effects) scored lowest at 0.4, while ICW (responsible for extracting precautions, warnings and interactions) reached 0.57. Table 2 summarizes mean score per question set, while Table 3 illustrates mean scores per field, presenting only those scoring below 1.

Independently of the content evaluation, all generated resource sets for the ten evaluation leaflets passed server-side $validate, confirming structural conformance only.

**Table 2:** Mean score per question set across leaflets, ordered alphabetically

Question Set	Overall Score	Profile based on
ICW	0,57	ClinicalUseDefinition
MK	0,92	MedicationKnowledge
MPD	0,94	MedicinalProductDefinition
ORG	1,00	Organization
UNDES	0,40	ClinicalUseDefinition

ICW Interactions Contraindications and Warnings, MK MedicationKnowledge, MPD MedicinalProductDefinition, ORG Organization, UNDES Undesirable Effects

**Table 3:** Mean score per field across leaflets ordered by score in descending order

Field	Overall Score	Source Profile
Admission number	0.90	MedicinalProductDefinition
Full name	0.90	MedicinalProductDefinition
Active ingredient/strength/doseform	0.90	MedicinalProductDefinition
Doseform	0.90	MedicationKnowledge
Dosing instructions	0.90	MedicationKnowledge
Dose and rate	0.90	MedicationKnowledge
Instructions on overdose	0.90	MedicationKnowledge
Mode of action	0.80	MedicinalProductDefinition
Strenght with unit	0.80	MedicinalProductDefinition
Maximum dose per intake	0.80	MedicationKnowledge
Instructions how to quit intake	0.80	MedicationKnowledge
Warnings	0.80	ClinicalUseDefinition
Additional instructions	0.70	MedicinalProductDefinition
Undesirable effects	0.40	ClinicalUseDefinition
Interactions with other medication	0.30	ClinicalUseDefinition
Precautions	0.20	ClinicalUseDefinition

## Discussion

This paper describes an automated pipeline that extracts medication leaflet information and structures it as FHIR resources with constraint-based validation against AMG § 16. The LLM-based question–answer pipeline performed best on clearly delimited leaflet sections. These sections are semantically narrow and show less overlap across categories, which supports targeted retrieval and explains why well-delimited fields reached high scores in the evaluation. Performance decreased for broader sections such as precautions, interactions, and undesirable effects, which are often heterogeneous, nested, and inconsistently structured across leaflets. In these sections, recurring errors were mainly observed as missing information and assignment errors, indicating difficulties in preserving subsection-level structure and mapping items to the correct subcategory. Complex sections can also contain semantically overlapping statements across categories, which increases ambiguity during extraction. For undesirable effects, missing information may be related to content being distributed across multiple leaflet subsections. Overall, these patterns suggest that failures in these sections are driven by heterogeneous leaflet structure and resulting model limitations in handling nested information. The findings support the approach as a proof of concept for well-delimited fields, but extraction from complex sections requires further improvement due to the safety-critical nature of medication information. The recurring error patterns provide concrete starting points to address these weaknesses. While additional prompt engineering may yield incremental gains, robust extraction from these sections will likely depend on addressing their underlying heterogeneity, for example through more capable models and more standardized leaflet structures.

Unlike document-centric ePI models using Composition containers, our resource-centric approach uses MedicinalProductDefinition for granular access. By narrowing the scope to medication leaflets, we operationalized AMG § 16 requirements through custom profiling and validation not found in broader ePI implementations. Beyond this Austrian context, the main methodological contribution is a profile-driven extraction workflow in which formally specified FHIR constraints, authored in FSH, inform prompt design and enable server-side validation. Transferability arises from reusing the same framework and pipeline logic, while adapting profiles, question sets, and mapping templates to the target regulation and document structure remains necessary.

The mapping mechanism automated FHIR resource creation for use cases involving manageable numbers of profiles and resources. In larger projects with substantially more resources and profiles, the approach may increase code complexity and lead to maintenance difficulties. This can be mitigated by centralizing recurring FHIR building blocks in shared helper functions and by defining straightforward key-to-element assignments per resource in a small mapping specification rather than implementing each assignment as separate code. As a key contribution to ongoing FHIR research, this work demonstrates that FHIR can be applied beyond patient-centric data to manage structured regulatory information such as medication leaflets.

## Limitations

FHIR profiles, the IG, and the question sets require ongoing maintenance. Regulatory updates and changes in model or library behavior may necessitate revising profiles, mapping logic, and prompts, but this long-term effort was not evaluated. Results are model- and prompt-dependent, so changes in model behavior may affect reproducibility and extraction quality. The scope was restricted to orally administered human medicines to reduce heterogeneity, limiting transferability to other dosage forms. Given the small sample (*n* = 10), findings are exploratory and suitable for proof-of-concept validation rather than generalizable performance claims. Manual field-level evaluation used the leaflet text as ground truth with binary scoring, but the absence of independent domain-expert annotation may lead to misclassification in clinically complex sections (e.g., interactions, contraindications). No formal usability evaluation was conducted, leaving end-user comprehensibility and acceptance unclear. The prototype used a locally deployed HAPI FHIR server, which limited deployment testing and reflects an infrastructural constraint rather than a methodological limitation. Finally, the IG lacks governance/versioning processes and relies largely on narrative text rather than coded terminologies, limiting semantic interoperability.

## Future Prospects

In future work, the identified limitations could be addressed by (i) increasing the number of evaluated leaflets and adding a pharmaceutical expert reference annotation to strengthen the manual field-level scoring, (ii) extending the extraction and mapping setup beyond orally administered medications to additional dosage forms, (iii) incorporate terminology bindings where feasible and (iv) conducting a usability evaluation with intended end users. A cloud-based deployment could further enable broader real-world testing across devices and locations.

## Data Availability

Medication leaflet data were retrieved from the publicly accessible AMPI ( [https://aspregister.basg.gv.at](https:/aspregister.basg.gv.at) ). The medication leaflets used in the evaluation are enumerated with their admission number in the provided repository ( [https://github.com/kathkirch/leaflet2fhir.git](https:/github.com/kathkirch/leaflet2fhir.git) ). The repository also contains the LLM responses for the evaluation and evaluation tables and samples of the generated FHIR resources. The Implementation Guide is fully reproducible from the repository ( [https://github.com/kathkirch/leaflet2fhir.git](https:/github.com/kathkirch/leaflet2fhir.git) ). The question sets are provided under /prompts/questionsets. Evaluation notebooks and sampling filters are in /eval/notebooks.
